# Construction of an m6A‐related lncRNA pair prognostic signature and prediction of the immune landscape in head and neck squamous cell carcinoma

**DOI:** 10.1002/jcla.24113

**Published:** 2021-11-16

**Authors:** Chongchang Zhou, Shumin Wang, Zhisen Shen, Yiming Shen, Qun Li, Yi Shen, Juntao Huang, Hongxia Deng, Dong Ye, Guowen Zhan, Jinyun Li

**Affiliations:** ^1^ Department of Otorhinolaryngology Head and Neck Surgery Ningbo Medical Center Lihuili Hospital Ningbo China; ^2^ Department of Otorhinolaryngology Head and Neck Surgery Lihuili Hospital affiliated to Ningbo University Ningbo China; ^3^ Department of Stomatology Huashan Hospital Fudan University Shanghai China; ^4^ Department of Otolaryngology Head and Neck Surgery Ningbo Yinzhou Second Hospital Ningbo China; ^5^ Department of Oncology and Hematology The Affiliated Hospital of Medical School of Ningbo University Ningbo China

**Keywords:** head and neck squamous cell carcinoma, immunotherapy, long non‐coding RNA, N6‐methylandenosine, prognosis

## Abstract

**Background:**

Mounting evidence indicates that aberrantly expressed N6‐methylandenosine (m6A) modification regulators and long noncoding RNA (lncRNA) influence the development of head and neck squamous cell carcinoma (HNSCC). However, the prognosis of m6A‐related lncRNA (mrlncRNA) in HNSCC has not yet been evaluated.

**Methods:**

We retrieved transcriptome, somatic mutation, and clinical information from The Cancer Genome Atlas database and established a differently expressed mrlncRNA (DEmrlncRNA) pair signature based on least absolute shrinkage and selection operator Cox regression and multivariate Cox analyses. Each sample's risk score was computed premised on the signature, which accurately classified patients into low‐ and high‐risk group by the cut‐off point. The signature was evaluated from the perspective of survival, clinicopathological characteristics, tumor mutation burden (TMB), immune cell infiltration, efficacy of chemotherapeutics, tumor immune microenvironment, and immune checkpoint inhibitor (ICI)‐related genes.

**Results:**

11 DEmrlncRNA pairs were identified and were used to construct the prediction signature. Kaplan–Meier plotter revealed a worse prognosis in high‐risk patients over low‐risk patients (log rank *p* < 0.001). According to multivariate Cox regression analysis, the hazard ratio of the risk score and 95% confidence interval of 1.722 and (1.488–1.992) (*p* < 0.001) were obtained. Furthermore, an increased risk score was associated with aggressive clinicopathological features, specific tumor immune infiltration status, increased expression of ICI‐related genes, higher TMB, and higher chemotherapeutics sensitivity (all *p* < 0.05).

**Conclusion:**

This research demonstrated that the signature premised on DEmrlncRNA pairs was an efficient independent prognostic indicator and may provide a rationale for research on immunotherapeutic and chemotherapeutics strategies for HNSCC patients.

## Graphical abstract

The m6A‐related lncRNA pair prognostic signature was evaluated from the perspective of survival, clinicopathological features, immune cell infiltration, tumor immune microenvironment, tumor mutation burden, efficacy of chemotherapeutics, and immune checkpoint inhibitor ‐related genes. The m6A‐related lncRNA pair signature was an efficient independent prognostic indicator and may provide a rationale for research on immunotherapeutic and chemotherapeutics strategies for HNSCC patients.

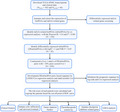

## 1 INTRODUCTION

Head and neck cancer is the seventh most frequent form of malignancy globally.^1^ Head and neck squamous cell carcinoma (HNSCC) is the most common pathological subtype, comprising over 90% of head and neck cancer patients.^2^ Approximately, one million new HNSCC cases are diagnosed worldwide yearly, which causes more than 14,000 deaths per year in the USA^3^ and more than 543,000 deaths per year worldwide.^4^ The risk factors contributing to HNSCC development include sustained exposure to tobacco and alcohol, viral infections by high‐risk oncogenic human papillomavirus (HPV), and familial inheritance.^5^ Due to the hidden physiological location of HNSCC, early diagnosis is difficult with most patients diagnosed at an advanced stage. Local recurrence, cervical node metastasis, and low therapeutic response rates to radiotherapy and chemotherapy are responsible for a persistently high mortality rate in advanced HNSCC patients.^6^ To improve the prognosis of HNSCC, it is urgent to understand the detailed molecular mechanisms underlying carcinogenesis of HNSCC and to identify reliable prognostic biomarkers that contribute to optimized personalized treatment strategies for HNSCC patients.

N6‐methylandenosine (m6A) modification is the commonest modification of noncoding RNAs (ncRNAs) and messenger RNAs (mRNAs).^7^ M6A is involved in numerous basic biological processes including RNA splicing, stability, translation efficiency, microRNA biogenesis, subcellular localization, and cell fate transition.^8^, ^9^, ^10^ M6A modification acts as a type of dynamic reversible process modulated by “readers” (binding proteins), “writers” (methyltransferases), and “erasers” (demethylases).^11^ *ZC3H13*, *RBM15*, *RBM15B*, *VIRMA*, *WTAP*, *METTL3*, *METTL14*, and *METTL16* have been shown to act as m6A methyltransferases,^12^ while *IGF2BP1*/*2*/*3*, *YTHDC1*, *YTHDC2*, *YTHDF1*/*2*/*3*, *HNRNPA2B1*, *HNRNPC*, and *RBMX* have been recognized as the “readers” of m6A and regulate mRNA metabolic activities.^13^, ^14^ *FTO*, *ALKBH3*, and *ALKBH5* were m6A demethylases and specifically remove m6A from target mRNAs.^15^, ^16^ Increasing evidence has demonstrated that dysregulated m6A modification is correlated with disorders of many biological processes and plays a crucial regulatory effect on tumor progression, prognosis, and resistance to radiotherapy.^17^ Arumugam et al.^18^ proposed that the genetic modifications of m6A regulatory genes are related to tumorigenesis and metastasis in HNSCC. Recently, Li et al.^19^ revealed that *YTHDC2* is a promising marker to predict prognosis and immune infiltration of HNSCC.

Long noncoding RNA (lncRNA) has been recognized as a kind of ncRNA with over 200 nucleotides in length.^20^ Increasing evidence has revealed that lncRNAs might act as promising biomarkers and potential targets to diagnose and treat cancer and are closely associated with immunity, proliferation, migration, apoptosis, and autophagy of cancer cells.^21^ Based on recent studies, the dysregulation of various types of lncRNAs in HNSCC has been reported to be associated with tumor progression, clinical stage, lymph node metastasis, and dismal prognosis.^22^ LINC00460 promotes epithelial–mesenchymal transition in HNSCC cells via mediating the entry of PRDX1 into the nucleus and has been proposed as a promising prognostic predictor and a possible target for cancer treatment in HNSCC.^23^ Kolenda et al.^24^ reported that ZFAS1 exhibits oncogenic capabilities and modulates vital processes related to EMT, cancer‐initiating cells, and metastases and could influence HNSCC patients’ clinical outcomes.

Although great progress has been made in identifying biomarkers for tumor prognosis, only less than 1% of identified biomarkers have been applied to clinical practice.^25^ Furthermore, most research has focused on a single cancer‐related biomarker. Based on recent studies, the modification of m6A is not just a pervasive mRNA modification but is also extensively present in lncRNAs.^26^, ^27^ Surprisingly, it has been reported that noncoding RNAs also exert a regulatory role in m6A modifications.^28^ Therefore, the combination of m6A modification and noncoding RNAs may represent an approach for the identification of synergistic cancer‐related biomarkers for clinical application. However, whether m6A‐related lncRNAs (mrlncRNAs) exert therapeutic effects and influence prognosis has yet to be fully explored in HNSCC. Therefore, the present study aimed to identify differentially expressed m6A‐related lncRNAs (DEmrlncRNAs) pairs in HNSCC, which were subsequently utilized to establish a signature to improve prognostic risk stratification and treatment decision‐making. Subsequently, associations between the risk score, clinicopathological variables, tumor mutation burden (TMB), immune cell infiltration, chemotherapeutic sensitivity, tumor microenvironment, and immune checkpoint inhibitor (ICI)‐related genes were systematically assessed to additionally examine the function of the proposed signature on clinical practice.

## 2 MATERIALS AND METHODS

### 2.1 Public data collection and processing

The RNA‐seq transcriptome data, mutation data, and associated clinical features of TCGA‐HNSC datasets were retrieved from the NCI’s Genomic Data Commons (https://portal.gdc.cancer.gov/, last accessed: 3 January 2021). In total, 502 primary HNSCC cases and 44 adjacent normal control cases were incorporated in the present study. Transcriptome data were retrieved as Fragments Per Kilobase Million (FPKM). Somatic mutation data from TCGA were retrieved in the mutation annotation file format, which was subjected to further analysis, extraction, and data visualization by applying the “maftools” package.^29^ The collected clinical characteristics of HNSCC patients included age, gender, grade, clinical stage, lymph node metastasis, T classification, overall survival (OS) status, and survival.

### 2.2 Data preparation and detection of mrlncRNAs in HNSCC patients

Extraction of expression matrixes of 22 m6A regulators from TCGA expression data was premised on earlier studies and included writers (*ZC3H13*, *RBM15*, *RBM15B*, *VIRMA*, *WTAP*, *METTL3*, *METTL14*, and *METTL16*), erasers (*ALKBH5*, *FTO*, and *ALKBH3*), and readers (*RBMX*, *HNRNPA2B1*, *HNRNPC*, *YTHDF3*, *YTHDF2*, *YTHDF1*, *IGF2BP3*, *IGF2BP2*, *IGF2BP1*, *YTHDC1*, and *YTHDC2*). The differential expression of m6A modulators in HNSCC tissues was obtained by comparison with normal tissues and was evaluated using the “pheatmap” and “limma” R package. The lncRNA annotation file of Genome Reference Consortium Human Build 38 Release 29 (GRCh38.p29) was retrieved from Ensembl (http://asia.ensembl.org) for annotation to distinguish mRNAs and lncRNAs in the TCGA database for subsequent analysis. Pearson correlation analysis was performed for m6A‐related genes and all lncRNAs using the “limma” R package to identify mrlncRNAs (with |Pearson R| >0.4 and *p* < 0.001). Then, DEmrlncRNAs in HNSCC tissues were contrasted with normal tissues using the “limma” R package with log fold change (logFC) > 1 and a false discovery rate (FDR) *p* < 0.05. The DEmrlncRNAs were sequentially and individually paired, and a 0 or 1 expression matrix was created using a previously reported method^30^ to eliminate batch effects due to the different platforms. If a DEmrlncRNA pair showed higher expression than a subsequent DEmrlncRNA, the value was assigned to 1. Conversely, if a DEmrlncRNA had lower expression than a subsequent DEmrlncRNA, it was given a value of 0. DEmrlncRNA pairs having a 0 or 1 expression level occupied over 20% of the aggregate number of pairs and were regarded as effective matches.

### 2.3 Development of the m6A‐related lncRNA pairs prognostic model

Firstly, univariable Cox regression analysis was employed to select DEmrlncRNA pairs correlated with the prognosis of HNSCC patients using “survival” R package with a *p* < 0.05. A total of 44 DEmrlncRNA pairs were obtained after screening. Secondly, the foremost DEmrlncRNA pairs for risk model construction were selected by using least absolute reduction and election operator (LASSO) Cox regression analysis. The performance of the LASSO algorithm was set with penalty coefficient tuning through a 10‐fold cross‐validation and a *p* < 0.05. The LASSO regression was run for 1000 cycles to exclude DEmrlncRNA pairs that may be highly correlated with other pairs. Finally, the coefficients were gotten by applying multivariate Cox regression analysis. “survival,” “survminer,” and “glmnet” were among the R packages used in these phases. The calculation of the risk score for all HNSCC patients was performed using the formula below:

$$\text{Risk}\;\text{score}=\sum_{i=0}^{n}\text{coefi}\times \text{DEmrlncRNA pair}$$

### 2.4 Validation of the prognostic of m6A‐related lncRNA pairs signature

The 1‐, 3‐, and 5‐year receiver operating characteristic (ROC) curves and the area under the curves (AUC) based on risk scores were generated to estimate the predictive effectiveness and were compared to other clinical characteristics such as sex, age, tumor grade, and stage. HNSCC patients were then classified in either low‐ or high‐risk groups premised on the inflection point of the maximum Youden index in the 1‐year ROC curve. Each HNSCC sample’s survival status and risk scores were visualized by risk curves and scatter plots. Differences in OS between the two groups were contrasted utilizing the log‐rank test and Kaplan–Meier (KM) survival. If the signature was evaluated as a potential independent prognostic predictor of HNSCC patients by employing multivariate and univariate Cox regression analyses. A prognosis nomogram for survival prediction based on the total points was established by including the independent prognostic indicators obtained from the multivariate Cox regression analysis.

### 2.5 Association analysis between the risk model and clinical features

To validate the clinical significance of the constructed signature, we utilized chi‐square tests to explore the association between the risk score and clinicopathological features of patients with HNSCC including gender, age, clinical stage, lymph node metastasis, T stage, and tumor grade. Differences in the risk score for different groups of clinicopathological features were detected utilizing Wilcoxon signed‐rank test. “survivalROC,” “survival,” “survminer,” “ggpubr,” and “ComplexHeatmap” were among the R packages utilized in these phases.

### 2.6 Correlation analysis between risk model, immune cell infiltration, and the tumor microenvironment

Using the CIBERSORT method, we computed the percentage of 22 immune cell kinds for each HNSCC specimen to determine whether there was a link between the risk score and immune cell infiltration. An algorithm with 1000 permutations was employed, and the subsequent analysis was performed including only samples with a *p* < 0.05.^31^ The calculation of the microenvironment score for each HNSCC sample (including stromal, immune, and estimate score) was performed using the “estimate” package of the ESTIMATE algorithm, an instrument used to forecast the tumor purity.^32^ The Wilcoxon signed‐rank test was employed to compare variations in immune infiltrating cell content and the microenvironment score for the high‐ and low‐risk groups, and the results were displayed in a violin plot. “e1071,” “preprocessCore,” “corrplot,” and “estimate” were among the R packages utilized in these analyses.

### 2.7 Correlation analysis between risk model and tumor mutation burden

Somatic variants data were annotated based on the hg19 reference genome followed by visualization utilizing the “GenVisR” package. The mutated genes in HNSCC were identified using the “maftool” package. Waterfall plots were used to display the topmost 20 commonly mutated genes in HNSCC patient samples belonging to both high‐ and low‐risk groups. The TMB was recognized as the quantity of coding, somatic, indels mutations, and base substitution for each megabase of the genome under investigation. To compute the TMB of each specimen, the overall number of mutations was divided by the size of the exome (38 megabases).^33^ The correlation between risk score and TMB was explored through the Wilcoxon signed‐rank test and Spearman’s correlation analysis. Furthermore, the KM plotter and the log‐rank test were employed to evaluate the OS of subgroups premised on risk score and TMB using “survival” and “survminer” packages.

### 2.8 Correlation analysis of risk models and chemotherapeutic sensitivity

The half inhibitory concentrations (IC50) of popular chemotherapy medicines for HNSCC patients were calculated using the “pRRophetic” package, for cisplatin, paclitaxel, docetaxel, and gemcitabine. The Wilcoxon signed‐rank test with a *p* < 0.05 was employed for the comparison of differences in IC50 values between low‐ and high‐risk groups.

### 2.9 Correlation analysis between risk score and the expression of ICI‐related genes

The differences in expression of the ICI‐related genes (including immunological‐checkpoint‐associated genes *IDO1*, *CTLA4*, *PDCD1*, *HAVCR2*, *CD274*, *LAG3*, and immune‐activation‐relevant genes *TNF*, *TBX2*, *IFNG*, *PRF1*, *GZMB*, *GZMA*, and *CD8A*) between the low‐ and high‐risk group were contrasted utilizing the Wilcoxon signed‐rank test. A two‐tailed *p* < 0.05 was recognized as statistically significant.

### 2.10 Gene set enrichment analysis

Gene set enrichment analysis (GSEA) (version 4.0.1) was utilized to find a possible molecular basis that differentiates the high‐ and low‐risk groups. MsigDB Collection (c2.cp.kegg.v7.1.symbols.gmt) was employed as the reference gene set. Following 1000 permutations, pathways having an FDR *p* < 0.05 were classified as substantially enriched.

## 3 RESULTS

### 3.1 Construction of a differentially expressed m6A‐related lncRNA pairs‐based prognostic signature

**FIGURE 1:**
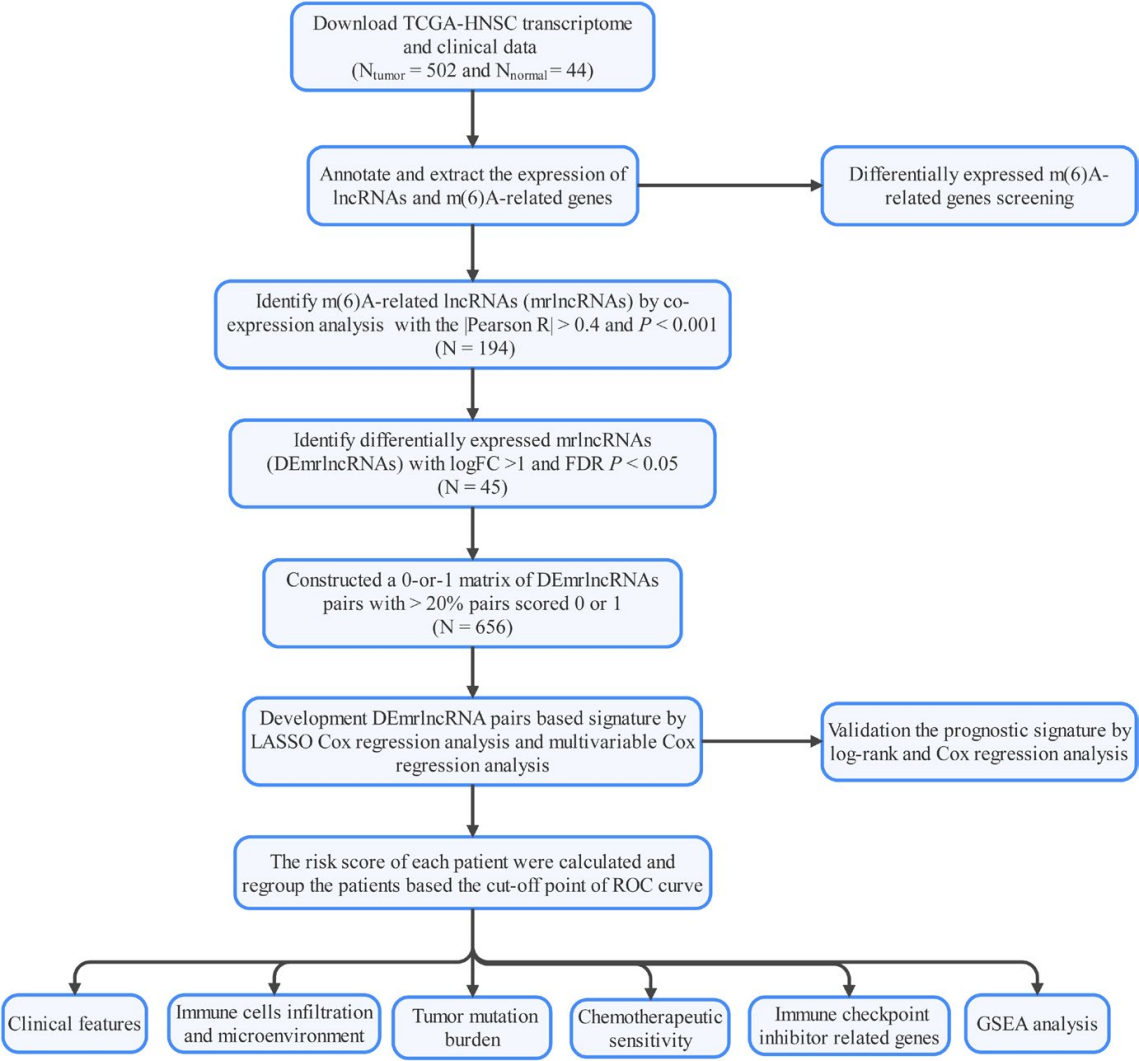
Flow diagram of the study. GSEA, gene set enrichment analysis; LASSO, least absolute shrinkage and selection operator; lncRNA, long noncoding RNA; ROC, receiver operating characteristic; TCGA, the cancer genome atlas

**FIGURE 2:**
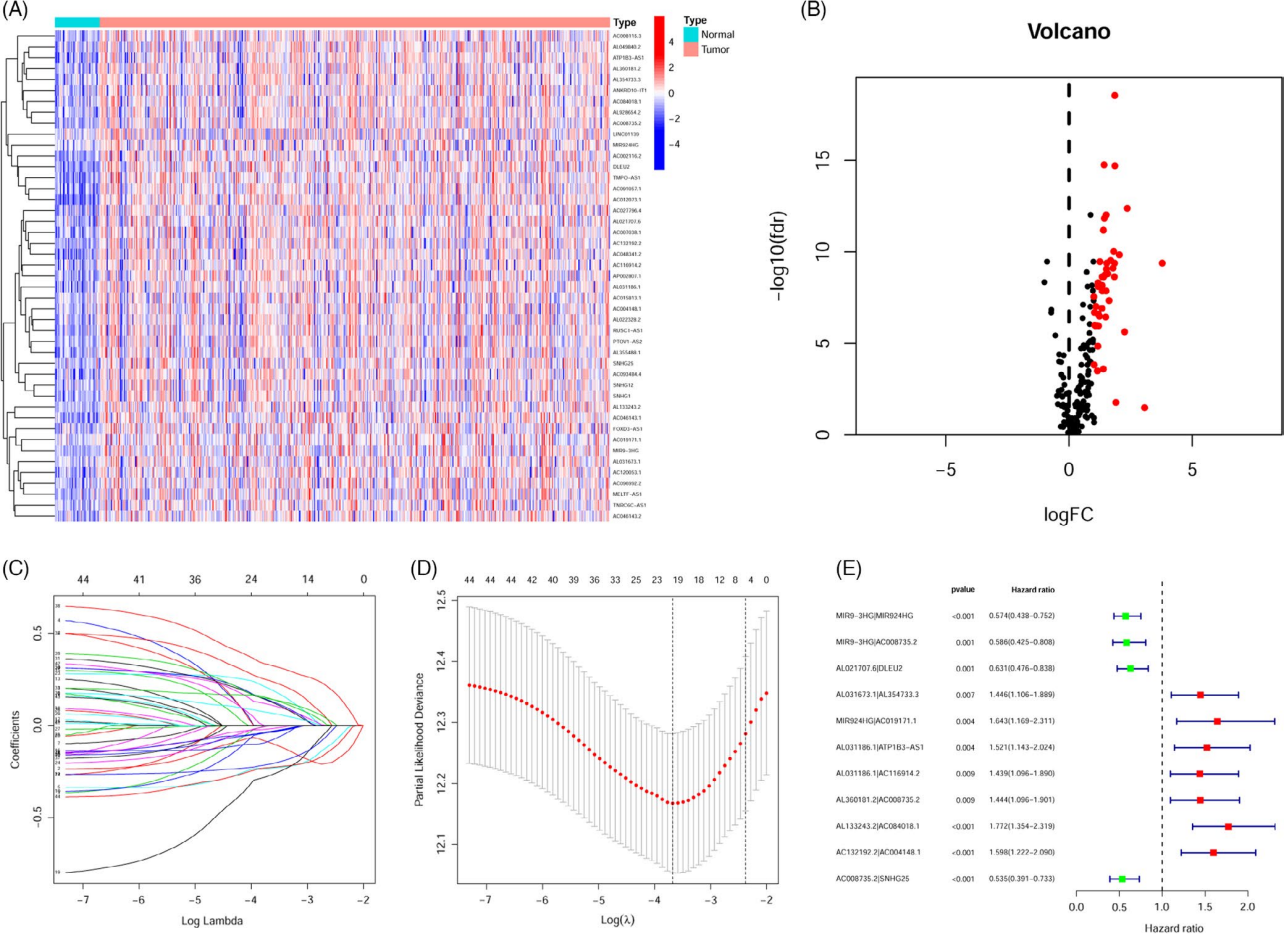
Development of a risk evaluation model using DEirlncRNA pairs. A, Heatmaps and (B) volcano plots deriving from the detection of differentially expressed m6A‐related lncRNAs (DEmrlncRNAs) determined by applying TCGA datasets and annotation by Ensemble. C, LASSO Cox regression analysis was conducted by computing the minimal criterion. D, Determination of penalties by 1000 rounds of cross‐validation optimal values for the parameters. E, Forest map showing the prognostic value of 11 DEmrlncRNA pairs, detected by Cox proportional hazard regression

Figure 1 shows the study flow used in this research. We retrieved the expression of 22 m6A RNA methylation modulators that included 8 writers, 3 erasers, and 11 readers, from the Genome‐wide expression matrix including 502 HNSCC and 44 normal samples from the TCGA‐HNSCC project. A heatmap (Figure S1A) was generated to visualize the expression pattern of 22 m6A modification modulators in HNSCC and normal samples. As shown in Figure S1B, we observed that most of the m6A modification regulators (*HNRNPA2B1*, *IGF2BP3*, *IGF2BP2*, *IGF2BP1*, *YTHDF3*, *YTHDF2*, *YTHDF1*, *ALKBH3*, *ALKBH5*, *FTO*, *RBM15*, *VIRMA*, *WTAP*, *METTL16*, *METTL14*, *METTL3*, *HNRNPC*, and *RBMX*) were remarkably upregulated in HNSCC samples comparing with normal control samples, whereas *YTHDC2* expression was decreased in the HNSCC samples. No considerable variation was observed for *RBM15B* (*p* = 0.18), *YTHDC1* (*p* = 0.3), or *ZC3H13* (*p* = 0.51) expression. The expression of 14,087 lncRNAs in HNSCC was extracted according to the annotation file (GRCh38.p29). We uncovered 194 mrlncRNAs using Pearson correlation analysis (Table S1). Next, an aggregate of 45 DEmrlncRNAs was detected (Figure 2A and Table S2), which were all upregulated in HNSCC (Figure 2B). After screening, 656 DEmrlncRNA pairs were constructed (Table S3). Univariate Cox analysis identified 44 significant prognostic DEmrlncRNA pairs (Table S4), which were incorporated in the LASSO Cox regression analysis (Figure 2C,D). Finally, 11 DEmrlncRNA pairs were identified and were used to build the prediction signature model weighted by the coefficients obtained using the multivariable Cox regression analysis (Table 1 and Figure 2E). Each specimen's risk scores were computed by applying the algorithm described above.

**TABLE 1:** Coefficients of DEmrlncRNA pairs used in the study

DEmrlncRNA pair	Coefficient
MIR9‐3HG|MIR924HG	−0.35588
MIR9‐3HG|AC008735.2	−0.59351
AL021707.6|DLEU2	−0.29937
AL031673.1|AL354733.3	0.360087
MIR924HG|AC019171.1	0.286023
AL031186.1|ATP1B3‐AS1	0.390976
AL031186.1|AC116914.2	0.243075
AL360181.2|AC008735.2	0.279647
AL133243.2|AC084018.1	0.353874
AC132192.2|AC004148.1	0.222544
AC008735.2|SNHG25	−0.32747

Abbreviation: DEmrlncRNA pair, differently expressed m6A‐related lncRNA pair.

### 3.2 Assessment of the DEmrlncRNA pairs‐based risk model

**FIGURE 3:**
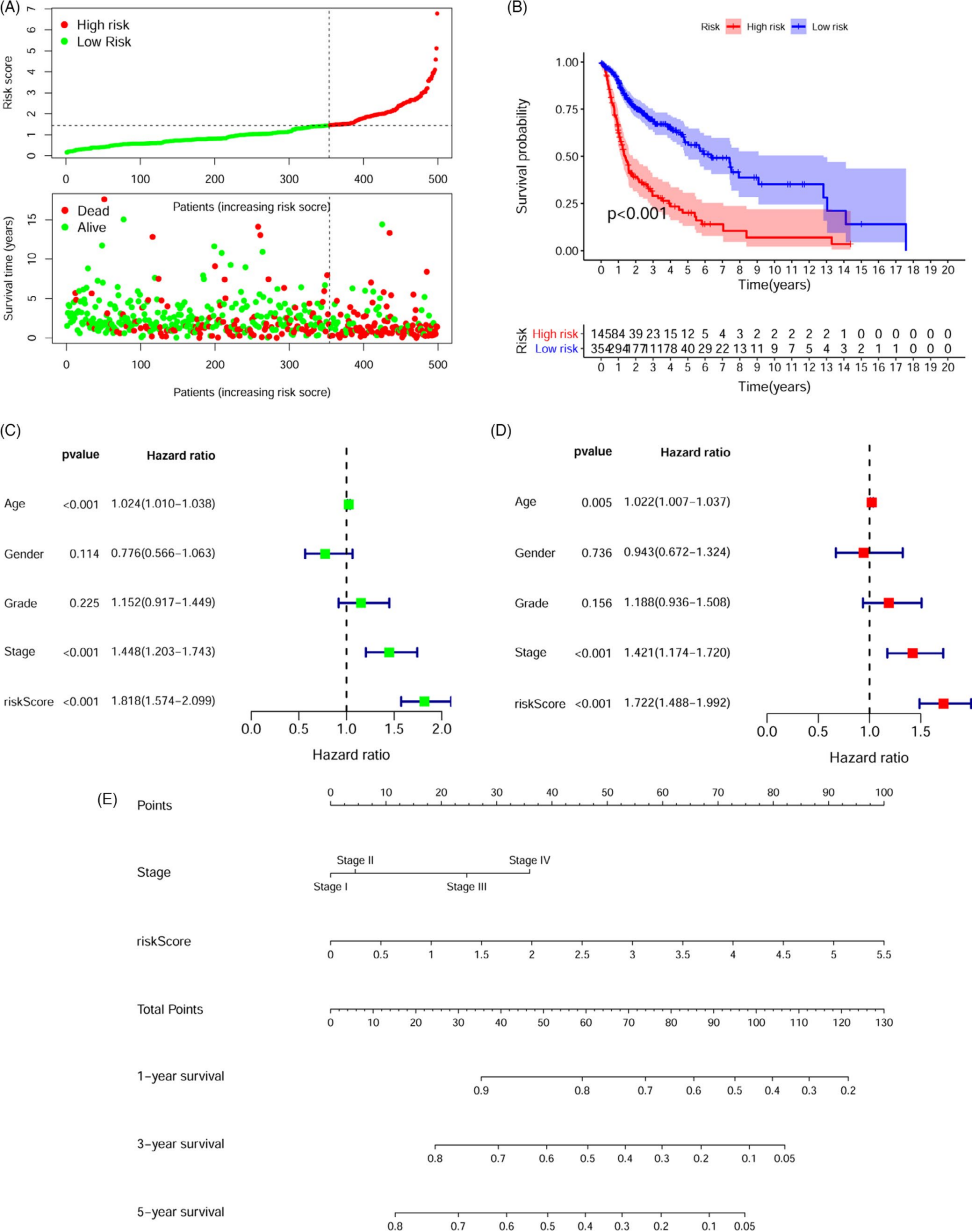
The prognostic significance of the DEmrlncRNA pairs signature. A, Distributions of risk scores and survival status of HNSCC patients in the TCGA dataset. B, KM curves of OS in the high‐ and low‐risk score groups. Patients in the high‐risk group experienced a briefer OS time. Univariate (C) and multivariate (D) analyses illustrated that risk score was an independent prognostic marker in the HNSCC patients. E, Nomogram premised on the clinical stage and risk score

**FIGURE 4:**
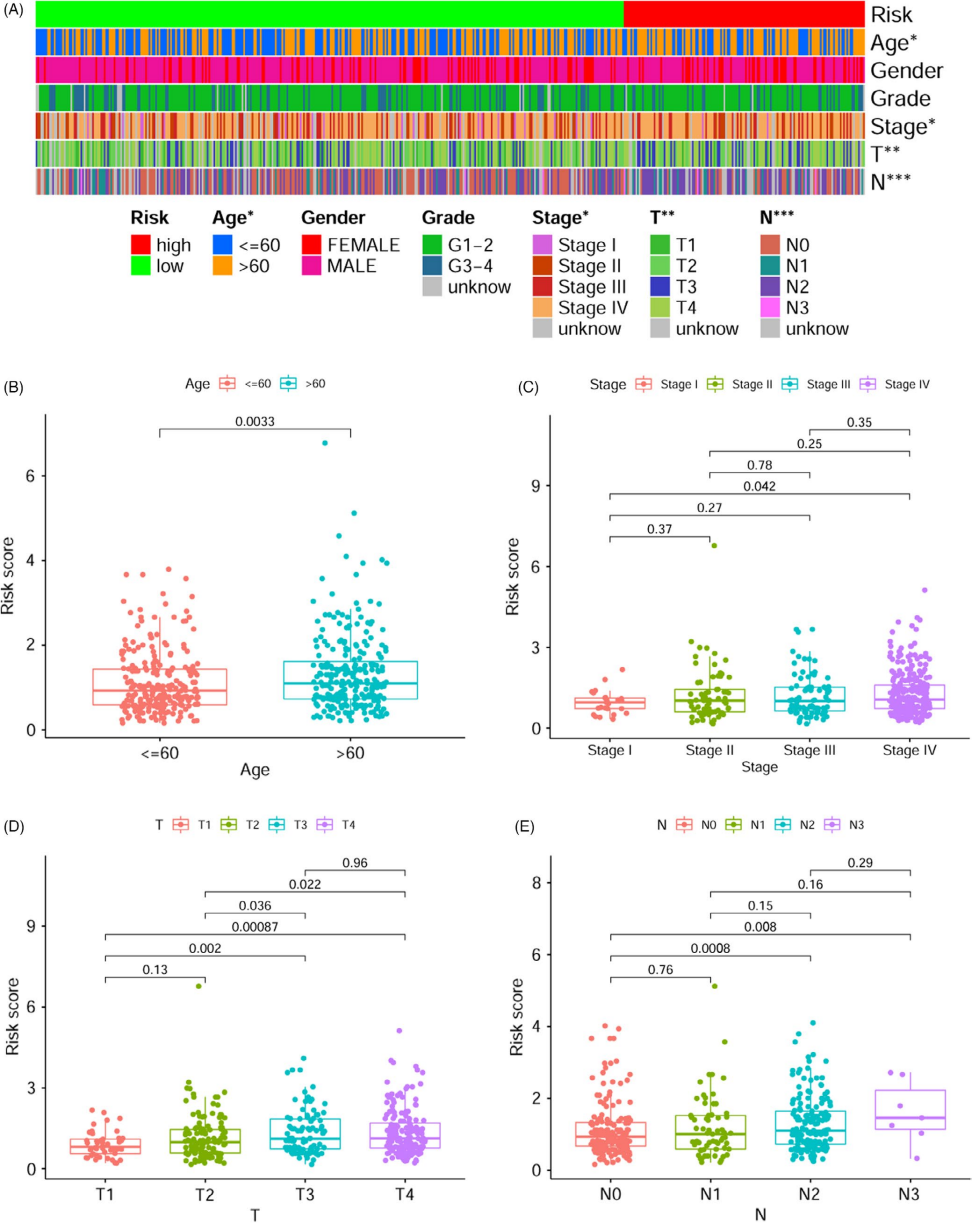
Relationship between clinical characteristics and the risk model. A strip chart (A) and the scatter diagram illustrated (B) age, (C) clinical stage, (D) T classification, and (E) lymph node metastasis were considerably linked to the risk score

The 1‐, 3‐, and 5‐year ROC curves of the risk score were charted to predict survival, and the AUC values obtained were 0.723, 0.733, and 0.717 (Figure S1A), respectively. These values were higher than the other clinical feature (age, gender, grade, and stage) based on the ROC curves (Figure S1B). Patients were classified into low‐ and high‐risk groups premised on the maximum Youden index obtained from the 1‐year ROC curve (Figure S2C, cut‐off point = 1.443). The scatter plot and risk curve illustrated that the death rates appeared to increase with increasing risk scores (Figure 3A). The KM curves and log‐rank test illustrated that the high‐risk group had a lower survival rate as opposed to the low‐risk group (Figure 3B, *p* < 0.001). Univariate (Figure 3C) and multivariate (Figure 3D) Cox analysis confirmed that the prognostic signature (HR = 1.722; 95% CI = 1.488–1.992; *p* < 0.001) and clinical stage (HR = 1.421; 95% CI = 1.174–1.720; *p* < 0.001) were independent prognostic indicators of poor OS for HNSCC patients. Thus, a nomogram (Figure 3E) was performed premised on the clinical stage and risk score, which contributed to define higher risk scores (range 0–100 points) for the poorer OS. After summing the points, the estimation of the survival likelihood was made by plotting a vertical line between the total points axis and the 1‐year, 3‐year, and 5‐year survival probability axes. Next, we explored the association between the clinicopathological variables and risk score utilizing the chi‐square test and Wilcoxon signed‐rank test. Both the strip chart (Figure 4A) as well as the scatter diagrams confirmed that age (Figure 4B), clinical stage (Figure 4C), T classification (Figure 4D), and lymph node metastasis (Figure 4E) were considerably linked to the higher risk scores.

### 3.3 The correlation between the immune cell infiltration, immune microenvironment, and ICI‐related genes

**FIGURE 5:**
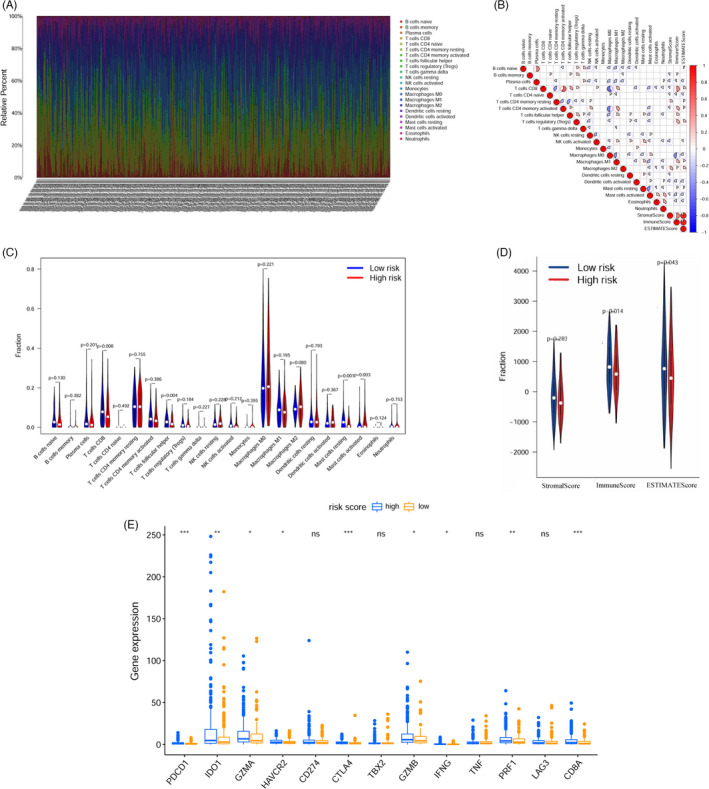
Approximation of immune cells infiltrating the immune microenvironment and expression of ICI‐related genes using the risk assessment model. A, Stacked bar chart displays the distribution of 22 immune cell types in each sample. B, Correlation matrix of immune cell proportions and tumor microenvironment score (immune score, stromal score, and ESTIMATE score). Blue signifies negative correlation, and red signifies positive correlation. C, Violin plot showing differentially infiltrated immune cells between high‐risk and low‐risk groups. D, Violin plot of the immune, stromal, and ESTIMATE scores in both the high‐ and low‐risk groups. E, The expression of *PDCD1*, *IDO1*, *GZMA*, *HAVCR2*, *CTLA4*, *GZMB*, *IFNG*, *PRF1*, and *CD8A* increased in the low‐risk group

We evaluated infiltrating immune cells utilizing the CIBERSORT algorithm and calculated the microenvironment scores using the ESTIMATE algorithm for each HNSCC specimen. Moreover, Figure 5A illustrates the composition of the 22 immune cell types for each specimen, which showed markedly different compositions. As shown in Figure 5B, the results from the correlation matrix illustrated that CD8 T cells were positively correlated with macrophages M1, activated NK cells, follicular helper T cells, activated CD4 memory T cells, Tregs, immune score, and the ESTIMATE score. Moreover, activated CD4+ memory T cells were found to have a positive link with M1 macrophages, resting NK cells, follicular helper T cells, immune score, and ESTIMATE score. Violin plot displayed that follicular helper T cells, CD8 T cells, and resting mast cells were more enriched in the low‐risk group; nevertheless, activated mast cells were discovered to be enriched in the high‐risk group (Figure 5C). Furthermore, we observed that both the immune and ESTIMATE scores were considerably decreased in the high‐risk group (Figure 5D). As ICIs have become increasingly important in the treatment of HNSCC in clinical settings, we evaluated the expression of ICI‐associated biomarkers in the two patient groups. High‐risk patients exhibited markedly elevated expression of *PDCD1*, *IDO1*, *GZMA*, *HAVCR2*, *CTLA4*, *GZMB*, *IFNG*, *PRF1*, and *CD8A* than low‐risk patients (Figure 5E).

### 3.4 Correlations between the risk model and tumor mutation burden

**FIGURE 6:**
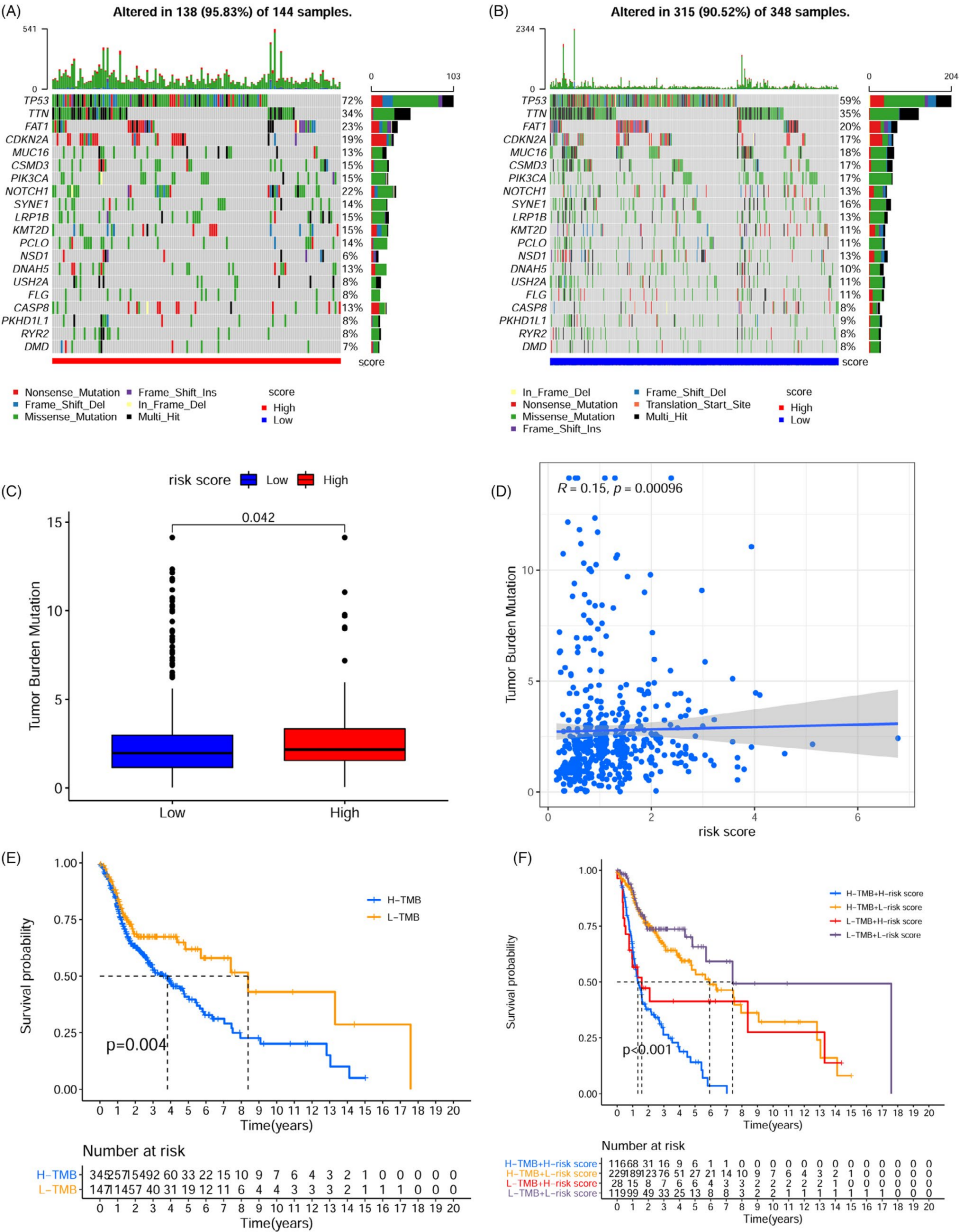
Correlation between the risk model and tumor mutation burden in HNSCC. The top 20 mutation genes in the high‐risk group (A) and low‐risk group (B) are shown in the waterfall plot. Different colors with specific annotations at the bottom indicate different mutation types. C, The TMB was higher in the high‐risk group compared with the low‐risk group. D, Scatter plots depicted a positive association between risk score and TMB. E, OS curves for high and low TMB cohorts (Log rank *p* = 0.004). F, OS curves for patients classified by both TMB and risk scores (Log rank *p* < 0.001)

To determine the mutation frequency of all genes, somatic mutation information was acquired (Table S5); the 5 topmost commonly mutated genes included *TP53* (63.62%), *TTN* (35.57%), *FAT1* (21.14%), *CDKN2A* (18.09%), and *MUC16* (17.28%). Mutation frequencies relative to the topmost 20 genes with the greatest mutation frequency in the high‐ (Figure 6A) and low‐risk groups (Figure 6B) are displayed in the waterfall plot. Given the significant clinical importance of the TMB, we calculated the TMB of each sample (Table S6) and compared the TMB of patients with low‐ and high‐risk groups to explore the inherent relationship between the TMB and risk scores. As illustrated in Figure 6C, high‐risk patients exhibited a considerably elevated TMB as opposed to low‐risk patients (*p* = 0.042). The risk score was strongly positively associated with the TMB, according to a Pearson correlation analysis (Pearson *r* = 0.15, *p* < 0.001, Figure 6D). Patients with higher TMB presented a shorter OS as opposed to those with a low TMB (log rank *p* = 0.004) (Figure 6E). According to the stratified subgroup survival analysis, the OS of the high TMB patients was poorer than low TMB patients in both the low‐ and high‐risk groups (Figure 6F, log rank *p* < 0.001).

### 3.5 Correlation analysis between risk score and the sensitivity of chemotherapeutic agents

**FIGURE 7:**
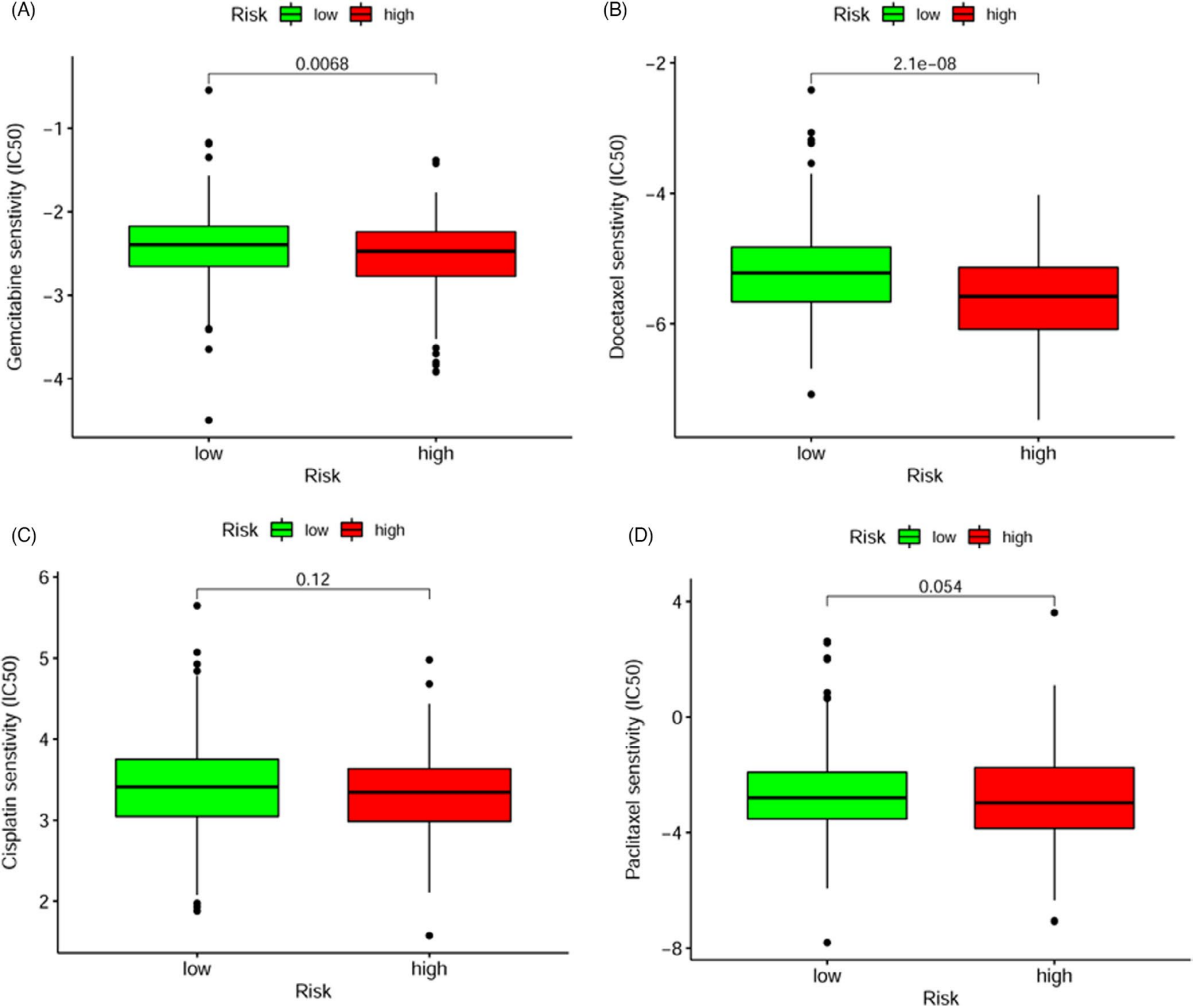
Correlation between risk models and chemotherapeutics in the HNSCC. A high‐risk score was correlated with a lower IC50 for gemcitabine (A) and docetaxel (B), whereas they were not significantly related to cisplatin (C) and paclitaxel (D)

Premised on the pRRophetic algorithm, the IC50 values for four common chemotherapeutic agents (docetaxel, gemcitabine, cisplatin, and paclitaxel) were predicted for the low‐ and high‐risk groups. The findings illustrated that gemcitabine (*p* = 0.007; Figure 7A) and docetaxel (*p* < 0.001; Figure 7B) had higher IC50 values in the low‐risk patients, indicating that the high‐risk score patients had a higher sensitivity to gemcitabine and docetaxel, while for cisplatin (*p* = 0.12; Figure 7C) and paclitaxel (*p* = 0.054; Figure 7D), these were not greatly correlated with higher IC50 values.

### 3.6 **GSEA identified the risk model for related signaling pathways**.

To identify a risk model associated with signaling pathways activated in HNSCC, the high‐ and low‐risk groups were compared applying GSEA analysis. As shown in Figure S3, ribosome (FDR *p* = 0.018) and proteasome (FDR *p* = 0.039) pathways were enhanced in the high‐risk group, whereas the Fc epsilon RI (FcεRI) signaling pathway (FDR *p* = 0.011), T cell receptor signaling pathway (FDR *p* = 0.023), GnRH signaling pathway (FDR *p* = 0.039), non‐small‐cell lung cancer (FDR *p* = 0.035), B cell receptor signaling pathway (FDR *p* = 0.049), phosphatidylinositol signaling system (FDR *p* = 0.046), aldosterone‐regulated sodium reabsorption (FDR *p* = 0.044), and the natural killer cell‐mediated cytotoxicity pathway (FDR *p* = 0.049) were enhanced in the low‐risk group.

## 4 DISCUSSION

Because of tumor heterogeneity and complicated carcinogenic mechanisms involved in HNSCC, the most applied TNM staging system fails to suitably interpret the prognosis of patients.^34^, ^35^ Hence, the identification of efficient prognostic biomarkers for HNSCC is a critical necessity. Accumulating research evidence has demonstrated that m6A modification is closely associated with cancer pathogenesis.^36^, ^37^ In this study, most m6A modification regulators were abnormally expressed in HNSCC except for *RBM15B*, *YTHDC1*, and *ZC3H13*. By altering the expression of certain lncRNAs, M6A regulators can keep some cancers malignant. Lan et al.^38^ revealed that KIAA1429 contributed to the progression of liver cancer via m6A‐dependent post‐transcriptional alteration of the lncRNA GATA3‐AS. The lncRNA GAS5 inhibits colorectal cancer development by interfacing with and activating YAP degradation and phosphorylation, which is adversely controlled by the m6A reader YTHDF3.^39^ However, it is currently unknown how m6A alteration affects HNSCC tumorigenesis and progression in a lncRNA‐dependent manner. Herein, based on 22 m6A regulators, 44 DEmrlncRNA pairs with prognostic value in HNSCC patients were filtered. We evaluated the DEmrlncRNA pairs with relatively higher or lower expression rather than investigating the specific expression of each single lncRNA, which showed the merit of clinical practicability.^30^, ^40^ Eleven DEmrlncRNA pairs were utilized to construct a reasonable prognostic signature for the prediction of the OS of HNSCC patients by the LASSO Cox regression analysis. Each HNSCC patient's risk score was computed premised on the coefficients of the prognostic model. Compared with other clinical attributes (stage, grade, sex, and age), the risk score for forecasting 1‐, 3‐, and 5‐year survival status of HNSCC had higher AUC values, which was also higher than the previous models.^41^, ^42^, ^43^ In addition, a higher risk score was considerably linked to older age, lymph node metastasis, and advanced stages, indicating that the risk score was more effective to stratify a patient's survival status. An ideal cut‐off point for model fitting was obtained using the Youden index rather than differentiating the risk merely premised on the median value. The Log‐rank test showed that HNSCC cases in the high‐risk group exhibited a considerably reduced OS as opposed to those in the low‐risk group, and further multivariate Cox regression analysis verified that the risk score was an independent risk indicator for OS of HNSCC patients. All these findings indicated that the prognostic signature identified herein was highly robust for prognosis prediction of HNSCC patients.

Immunotherapy is currently an area of active development in HNSCC.^44^ Although recent studies have indicated that the TME plays a critical role in immunotherapy,^45^, ^46^ the precise mechanisms involved are unclear. Thus, further investigation into the role of the TME is required to improve outcomes relying on immunotherapy. The TME of HNSCC comprises stromal cellular elements, immune cells, and tumor cells.^47^ Immune cells within the TME exert a significant effect on tumorigenesis. Immune cell dysfunction may exert tumor‐antagonizing or tumor‐promoting activity through a variety of mechanisms.^48^ In this research, we calculated the degree of infiltrating immune cells and microenvironment scores using CIBERSORT and ESTIMATE algorithms, respectively. Moreover, we observed that HNSCC samples having a low‐risk score were associated with increased CD8 T cell and follicular helper T cell infiltration and the immune score. GSEA analysis also revealed that the natural killer cell‐mediated cytotoxicity pathway, T cell receptor signaling pathway, B cell receptor signaling pathway, and the Fc epsilon RI signaling pathway were enhanced in the low‐risk group. A myriad of evidence has revealed that the presence of significant T cell infiltrates is linked to improvement in patient prognosis across several human malignancies^49^ and these infiltrates determine the probability of therapeutic response to cancer immunotherapies.^49^, ^50^ The Keynote‐001 study shows that pembrolizumab achieved a better response in non‐small‐cell lung cancer patients having greater CD8+ T cell infiltration than patients with reduced CD8+ T cell infiltration.^51^ Thus, it follows that the low‐risk HNSCC group presented variable T cell infiltration and presented a better prognosis.

As the most important immunotherapy drugs, ICIs—mainly represented by anti‐CTLA4 and anti‐PD antibodies—have shown an unexpected antitumor activity and have improved the prognosis in various types of cancer.^51^ In HNSCC, the anti‐PD‐1 antibodies pembrolizumab and nivolumab have received approval from the USA Food and Drug Administration and European Medicine's Agency for platinum‐refractory/relapsed or metastatic patients.^52^, ^53^ Supported by the findings of the phase III Keynote 048 clinical trial, pembrolizumab has been gained approval from both the EMA and FDA in the first‐line setting for either monotherapy or combination therapy with chemotherapy depending on the status of tumor expression of programmed cell death protein ligand‐1 (PD‐L1). Pembrolizumab treatment enhanced the survival of a subset of HNSCC patients.^54^ Nevertheless, it is crucial to identify markers that improve the selection of patients who could achieve better therapeutic responses due to the associated serious adverse reactions and high cost of treatment with ICIs. Thus, the association between the ICI‐related genes expression and the prognostic model was explored. High‐risk patients exhibited markedly higher expression of *PDCD1*, *IDO1*, *GZMA*, *HAVCR2*, *CTLA4*, *GZMB*, *IFNG*, *PRF1*, and *CD8A* as opposed to that of low‐risk patients, showing that high‐risk patients might show better responses and achieve better outcomes when receiving ICI treatment.

Extensive research has demonstrated that the TMB is associated with immune cell infiltration and might be responsible for the heterogeneous clinical responses to immunotherapy.^55^, ^56^ Thus, we examined the connection between the TMB and risk model and determined that patients having a high‐risk score also exhibited a considerably higher TMB as opposed to those with a low‐risk score. The Spearman correlation analysis determined that the risk score has a considerably positive association with the TMB. In addition, a previous study by Lan et al.^57^ revealed that HNSCC patients having a lower TMB presented a better prognosis than patients with a higher TMB, and the TMB itself might influence CD4+ T cell and B cell infiltration status. The results of our survival analysis are in line with these findings. Further stratified subgroup survival analysis illustrated that patients with low TMB exhibited a prolonged OS in both low‐ and high‐risk groups. A high TMB is indicative of the accumulation of somatic mutations in tumors, which leads to the exposure of more neoantigens^58^; thus, we speculated that the high‐risk group having a higher TMB would also present an enhanced immune response. Furthermore, we determined the IC50 values for four common chemotherapeutic agents used for treating HNSCC. Our results illustrated that the high‐risk group would have a greater sensitivity to gemcitabine and docetaxel treatment, and thus the signature constructed in the current study could act as a potential predictor for chemosensitivity and might result in the development of more precise chemotherapy.

There are, however, some limitations in this study that should be considered. Firstly, the number of patients used to construct the signature was relatively inadequate because it comprised only cases downloaded from the TCGA database. Secondly, our findings relied only on bioinformatics analysis and still need to be verified by large‐sample clinical trials containing survival information in future. Thirdly, the current investigation assessed the relationship between a risk signature and immune landscape and predicted the effects of immunotherapy and chemotherapy. The findings demand confirmatory clinical trials with larger HNSCC cohorts receiving immunotherapy and chemotherapy.

In conclusion, we constructed a DEmrlncRNA signature that could independently forecast prognosis in HNSCC patients without analyzing individual lncRNA expression levels. Furthermore, this signature might be used to reliably identify individuals who will stand to gain from immunotherapy or chemotherapy, which will contribute to the development of individualized and precise treatment for HNSCC patients.

## CONFLICTS OF INTEREST

The authors declare there are no financial or other conflicts of interest associated with this study.

## AUTHOR CONTRIBUTIONS

The research was designed by CZ, GZ, and JL. The data analysis from the public database was made by SW and Z. QL, YS, DY, and HD took part in data analysis. CZ was in charge of writing the draft. All authors reviewed the manuscript.

## Supporting information

Fig S1

Fig S2

Fig S3

Table S1

Table S2

Table S3

Table S4

Table S5

Table S6

## Data Availability

The data that support the findings of this study are openly available from The Cancer Genome Atlas (TCGA) program at https://portal.gdc.cancer.gov/.
