# Supplementary figures and images for: Construction of an m6A‐related lncRNA pair prognostic signature and prediction of the immune landscape in head and neck squamous cell carcinoma
Source: J Clin Lab Anal. 2021 Nov 16;36(1):e24113. doi: 10.1002/jcla.24113 (PMC8761472; doi:10.1002/jcla.24113)

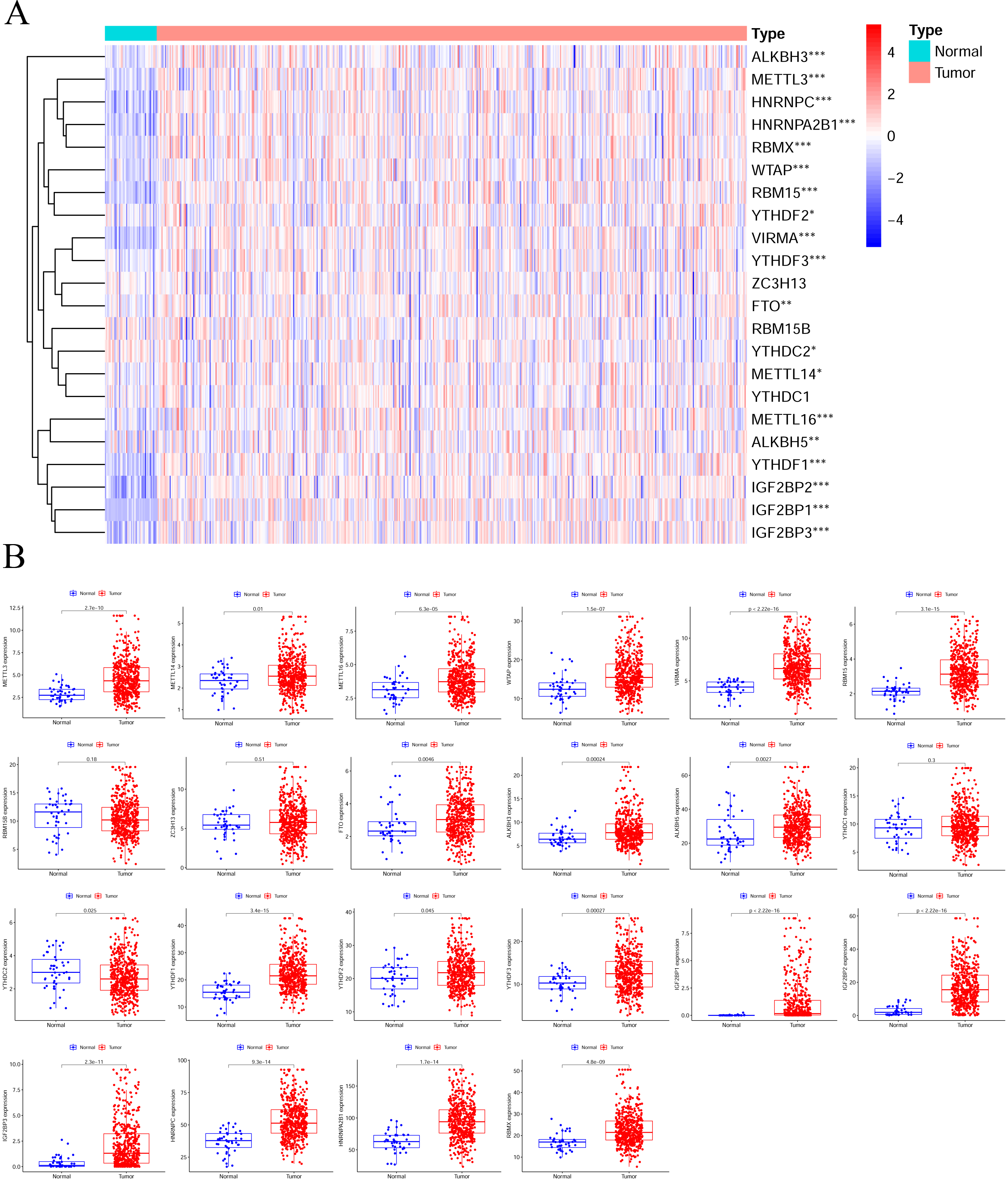

Supplement: Supplementary file 1 — Fig S1 [file JCLA-36-e24113-s004.tif]

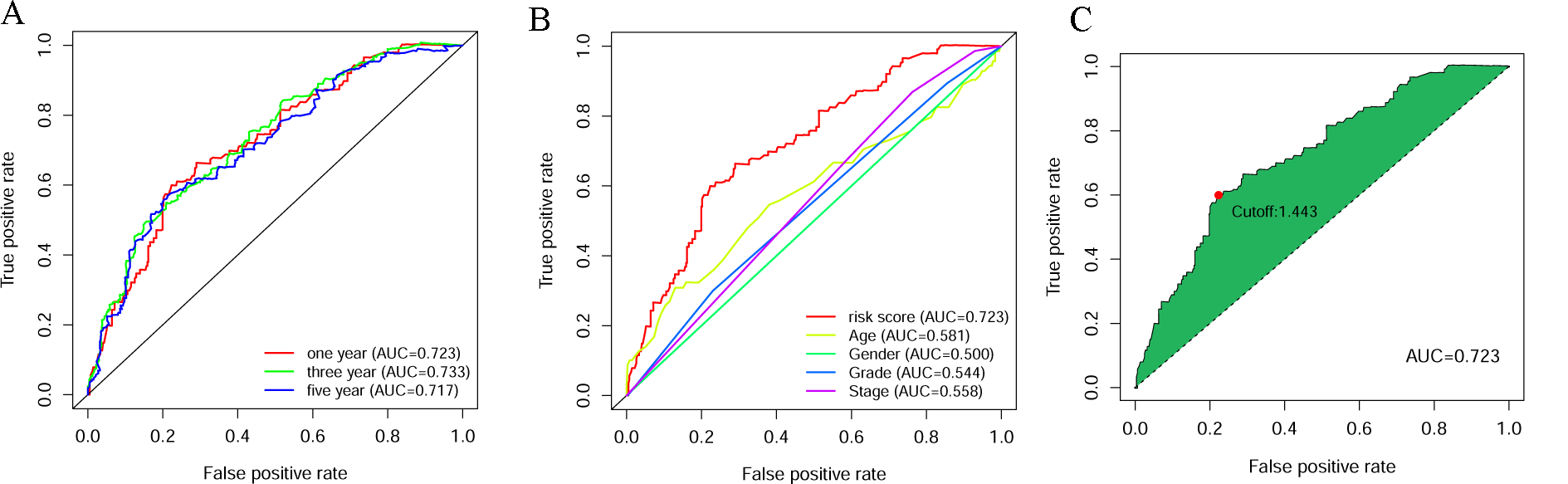

Supplement: Supplementary file 2 — Fig S2 [file JCLA-36-e24113-s002.tif]

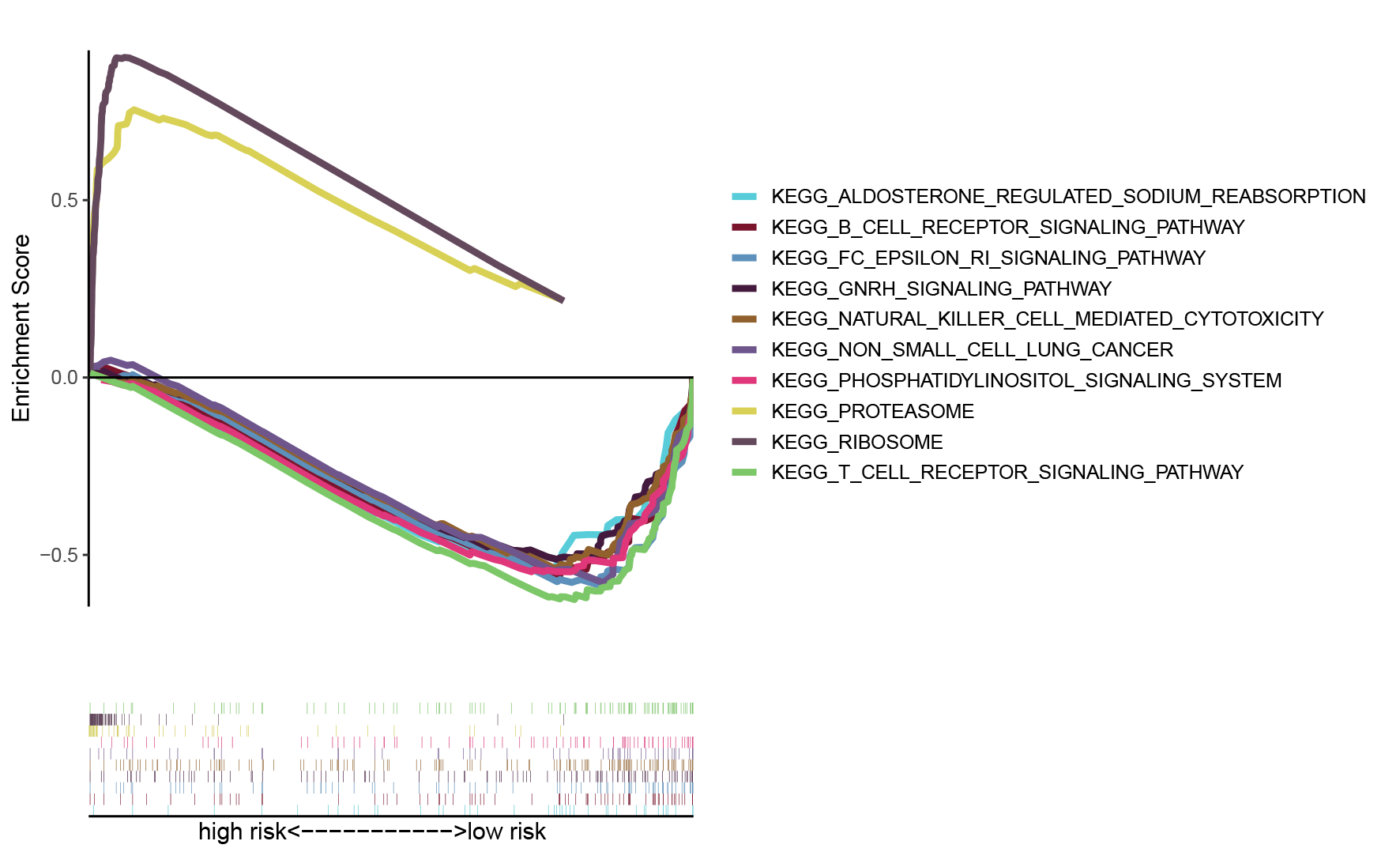

Supplement: Supplementary file 3 — Fig S3 [file JCLA-36-e24113-s009.tif]
